# Using a single question to assess physical activity in older adults: a reliability and validity study

**DOI:** 10.1186/1471-2288-12-20

**Published:** 2012-02-28

**Authors:** Dawn P Gill, Gareth R Jones, Guangyong Zou, Mark Speechley

**Affiliations:** 1 National Alzheimer's Coordinating Center, Department of Epidemiology, University of Washington, Seattle, WA, USA; 2 School of Health and Exercise Sciences, University of British Columbia, Kelowna, BC, Canada; 3 Department of Epidemiology and Biostatistics, Schulich School of Medicine and Dentistry, University of Western Ontario, London, ON, Canada; 4 Robarts Clinical Trials of Robarts Research Institute, Schulich School of Medicine and Dentistry, University of Western Ontario, London, ON, Canada

**Keywords:** Physical activity, self-report, single-item measure, assessment, validity, reliability, older adults

## Abstract

**Background:**

Single-item physical activity questions provide a quick approximation of physical activity levels. While recall questionnaires provide a more detailed picture of an individual's level of physical activity, single-item questions may be more appropriate in certain situations. The aim of this study was to evaluate two single-item physical activity questions (one absolute question and one relative question) for test-retest reliability, convergent validity, and discriminant validity, in a sample of older adults.

**Methods:**

Data was obtained from the Project to Prevent Falls in Veterans, a fall risk-factor screening and modification trial. One question measured absolute physical activity (seldom, moderately, vigorously active) and one measured relative physical activity (more, about as, less active than peers). Test-retest reliability was examined using weighted Kappa statistics (κ) in a sample of 43 subjects. Validity was assessed using correlation coefficients (*r*) in participants who received clinical assessments (n = 159).

**Results:**

The absolute physical activity question was more reliable than the relative physical activity question (κ = 0.75 vs. κ = 0.56). Convergent validity, however, was stronger for the relative physical activity question (*r *= 0.28 to 0.57 vs. *r *= 0.10 to 0.33). Discriminant validity was similar for both questions. For the relative physical activity question, there was moderate agreement when this question was re-administered seven days later, fair to moderate/good associations when compared with indicators of physical function, and little to no associations when compared with measures hypothesized to be theoretically not related to physical activity.

**Conclusions:**

The relative physical activity question had the best combination of test-retest reliability, convergent validity and discriminant validity. In studies requiring a measure of physical activity, where physical activity is not the primary focus and more detailed measures are not feasible, a single question may be an acceptable alternative.

## Background

When selecting measures for a study, investigators usually need to strike a balance among several factors such as required sample size, the level of detail needed, the resources available, and the burden posed by their measurement protocol on research participants. In particular, for a given research budget, there is typically a trade-off between measurement detail and sample size [1].

Each of these issues is present in the study of physical activity (PA) in older adults. Regular PA assists with maintaining independence and preventing disability among older adults and it is associated with a decreased risk of morbidity and all-cause mortality [2]. PA is defined as "any bodily movement produced by skeletal muscles that results in energy expenditure" [3]. PA is a complex behavioral construct that can be categorized and quantified in many ways. For example, PA can be broken down into routine activities such as housework, and those done for exercise, such as swimming. Each specific type of PA can be quantified in terms of frequency, intensity, and duration [4].

These complexities are reflected in the many methods used to measure PA or related energy expenditure in older adults [4-7]. Measurement of PA can be categorized into direct and indirect methods. Direct methods are defined as those that measure movement as it occurs and indirect methods provide indicators of PA and energy expenditure [5]. Examples of direct methods include motion sensors, such as pedometers, accelerometers, and Global Positioning Systems, whereas indirect methods include daily PA records or log books and self-report questionnaires.

There are a number of recall questionnaires that have been used in older adult populations, with varying degrees of evidence for reliability and validity [8]. While these questionnaires provide a more detailed picture of an individual's PA, global questionnaires or single-item questions may be favored in certain situations. When investigators have a choice of questions, they might compare the evidence of validity and reliability in making their selection. Reliability and validity results from existing single-item PA questions [9-12] indicate a need to evaluate additional single-item questions as possible measures of PA under certain conditions (i.e., when PA is not the primary focus of a study but a quick approximation of activity levels is of interest as a covariate or possible confounding factor, when the sample size is large, when resources are limited, and when more complex methods would add to respondent burden).

Similar research has been done with general health measures. A previous study found that two single-item general self-rated health measures showed good measurement properties when compared to a multi-item instrument, thus providing a less burdensome alternative [13]. In that study, researchers compared "standard" and "comparative" versions of general self-rated health measures, where the comparative version referred to a question that had respondents compare their general health to a reference group. Findings indicated that both questions represented reasonably similar assessments of health. Another study in this area, which compared three different single-item questions of self-rated health (two "standard" questions and one "comparative" question) found similar results [14].

In a previously completed fall risk factor modification trial, two single-item questions of PA were included, both intended to easily classify activity levels of participants. Similar to the self-rated health literature, one question was a "standard" measure and one was a "comparative" measure. Specifically, one question measured absolute PA (seldom, moderately, vigorously active) and the other measured relative PA (more, about as, less active than peers). Using the self-rated health literature as a model, since both PA questions have the same intent (i.e., to quickly classify PA levels), it is of interest to determine if properties of reliability and validity are similar between these questions and whether they could be used interchangeably. Thus, the aim of this study was to evaluate the test-retest reliability, convergent validity and discriminant validity of an absolute PA question and a relative PA question, in a sample of community-dwelling older adults.

## Results

The characteristics of participants who took part in the reliability sub-study are described in detail elsewhere [15]. Briefly, the mean age was 79 (standard deviation (SD) 2.9) years and approximately one-half were male.

**Figure 1:**
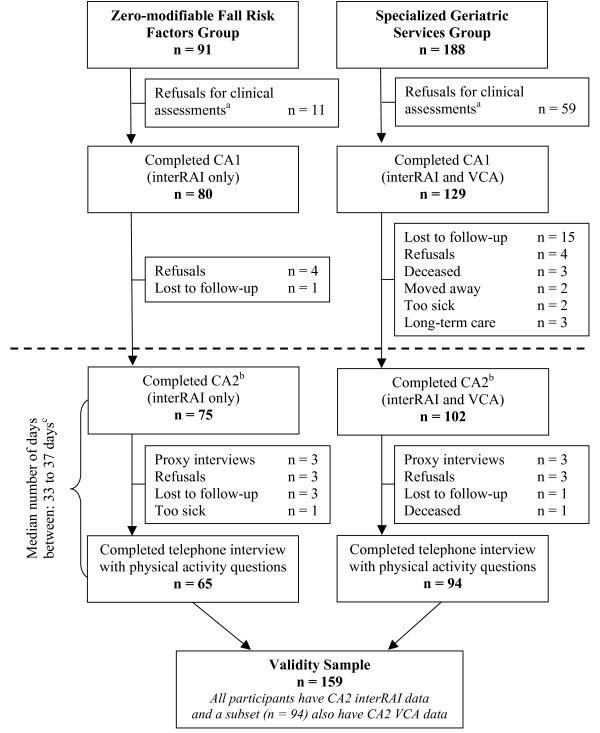
**Formation of validity sample from the Project to Prevent Falls in Veterans**. The dashed line indicates the point in the larger study where measures of interest for the present study begin. ^a ^Clinical assessments were not required to be part of the larger study. ^b ^Validation measures used in the present study were taken from CA2. ^c ^CA2 and telephone interviews were completed as close together in time as possible. The median number of days was 37 days for all participants (n = 159) and 33 days for the subset who also had VCA data (n = 94). Abbreviations: CA1 = first clinical assessment; interRAI = interRAI Community Health Assessment (earlier version); VCA = Veterans' Comprehensive Assessment; CA2 = second clinical assessment.

For the validity sample, the mean age was 80 (SD 3.9) years, and close to two-thirds were males. Other characteristics of participants included in the validity sample are presented in Table 1. About 20% self-reported fair or poor health, 38% reported one or more falls in the past 12 months, 49% reported that their memory was worse than five years ago, 15% reported being seldom active and 12% reported that they were less active compared to their peers. In comparison to women, men were older (mean age 81 years, SD 3.5), and a slightly higher percentage reported fair or poor health, one or more falls in the past month and worse memory compared to five years earlier. Men and women provided similar responses in regard to their PA compared to their peers. The median time between administration of validation measures and PA questions ranged between 33 days for the subset of participants who had been administered both the earlier version of the interRAI Community Health Assessment (interRAI) and Veterans' Comprehensive Assessment (VCA), and 37 days when considering all participants in the validation sample (also see Figure 1).

**Table 1:** Participant characteristics (validity sample)^a^

	Total (N = 159)	Women (n = 58)	Men (n = 101)
	
Characteristic	n (%)	n (%)	n (%)
Activity level			
Seldom active	24 (15.1)	7 (12.1)	17 (16.8)
Moderately active	96 (60.4)	41 (70.7)	55 (54.5)
Vigorously active	38 (23.9)	10 (17.2)	28 (27.7)

Activity level compared to peers			
Less active	19 (11.9)	7 (12.1)	12 (11.9)
About as active	61 (38.4)	21 (36.2)	40 (39.6)
More active	78 (49.1)	30 (51.7)	48 (47.5)

Participant Status			
Veteran	103 (64.8)	3 (5.2)	100 (99.0)
Caregiver	56 (35.2)	55 (94.8)	1 (1.0)

Finances at end of month			
Just enough money	35 (22.0)	11 (19.0)	24 (23.8)
Money left over	123 (77.4)	47 (81.0)	76 (75.2)

1+ falls in past 12 months	61 (38.4)	17 (29.3)	44 (43.6)

1+ injurious falls in past 12 months	20 (12.6)	8 (13.8)	12 (11.9)

Memory compared to 5 years earlier			
Worse	78 (49.1)	25 (43.1)	53 (52.5)
About the same	79 (49.7)	32 (55.2)	47 (46.5)
Better	2 (1.3)	1 (1.7)	1 (1.0)

Self-rated health			
Poor	4 (2.5)	2 (3.5)	2 (2.0)
Fair	28 (17.6)	7 (12.1)	21 (20.8)
Good	62 (39.0)	24 (41.4)	38 (37.6)
Very good	48 (30.2)	15 (25.9)	33 (32.7)
Excellent	17 (10.7)	10 (17.2)	7 (6.9)

^a^All participants included had a second clinical assessment (CA2) and were evaluated with the earlier version of the interRAI Community Health Assessment (interRAI). A subset (n = 94) receiving a CA2 were also evaluated with the Veterans' Comprehensive Assessment (VCA)

Percentages were calculated excluding those with missing values

Results indicated that the absolute PA question had better test-retest reliability than the relative PA question. The weighted kappa value for absolute PA was 0.75 (95% confidence interval (CI): 0.60 to 0.91) whereas for relative PA, the weighted kappa value was 0.56 (95% CI: 0.30 to 0.82). For the absolute PA question, the weighted kappa value indicated substantial agreement whereas for relative PA, the weighted kappa value indicated agreement in the moderate range.

The validation results for both the absolute and relative PA questions are presented in Table 2. For both PA questions, correlation coefficients were in the expected directions according to the type of validity being assessed. For the relative PA question, there was greater contrast between values obtained for convergent validity and discriminant validity. For absolute PA, correlations with convergent validation measures ranged from 0.10 (95% CI: 0.00 to 0.26) to 0.33 (95% CI: 0.19 to 0.49), indicating relationships ranging from little to a fair degree of association. For relative PA, correlations were consistently higher, with most comparisons indicating fair to moderate or good associations. Specifically, correlations ranged from 0.28 (95% CI: 0.15 to 0.44) to 0.57 (95% CI: 0.38 to 0.78). The total score on the balance assessment, limiting PA due to fear of falling, and all of the gait measures (unsteady gait, gait-path, gait-trunk, and gait abnormality) had stronger positive correlations with relative PA, compared to absolute PA.

**Table 2:** Convergent and discriminant validity results for PA questions

	**Absolute Question**^**a**^	**Relative Question**^**b**^
**Validation Measure**	**r (95% CI)**^**c**^	**r (95% CI)**^**c**^

*Convergent validity*		

Total score on Berg Balance Scale (*n *= 158)	0.31 (0.17, 0.45)	0.48 (0.35, 0.59)

Unsteady gait (*n *= 152)	0.24 (0.12, 0.40)	0.40 (0.26, 0.56)

Gait-path (*n *= 93)	0.32 (0.17, 0.53)	0.57 (0.38, 0.78)

Gait-trunk (*n *= 94)	0.29 (0.15, 0.50)	0.42 (0.25, 0.63)

Gait-abnormality (*n *= 93)	0.26 (0.00, 0.47)	0.36 (0.20, 0.57)

Lower extremity weakness (*n *= 92)	0.21 (0.00, 0.43)	0.37 (0.21, 0.58)

Postural stability (*n *= 94)	0.17 (0.00, 0.38)	0.33 (0.17, 0.54)

Walking ability (*n *= 158)	0.33 (0.19, 0.49)	0.42 (0.28, 0.58)

Housework difficulty *(n *= 158)	0.28 (0.16, 0.45)	0.34 (0.20, 0.50)

Limits activity-fear of falling (*n *= 155)	0.10 (0.00, 0.26)	0.28 (0.15, 0.44)

Pain affects mobility (*n *= 90)	0.28 (0.00, 0.50)	0.34 (0.18, 0.55)

*Discriminant validity*		

Home hazards (*n *= 152)	0.04 (0.00, 0.19)	0.02 (0.00, 0.12)

Vision (*n *= 157)	0.05 (0.00, 0.21)	0.07 (0.00, 0.23)

Skin problems (*n *= 157)	0.13 (0.00, 0.29)	0.15 (0.00, 0.32)

Hearing deficit (*n *= 94)	0.29 (0.15, 0.50)	0.24 (0.00, 0.44)

^a ^Response categories: i) seldom active; ii) moderately active; or iii) vigorously active.

^b ^Response categories: i) less active; ii) about as active; or iii) more active.

^c ^Specific type of correlation coefficient (*r*): Spearman's rho (total score on Berg Balance Scale) and Cramer's V (all other validation measures)

Validation measures and PA questions were ordered so that higher scores indicated better functioning or higher PA levels.

Abbreviations: CI = Confidence Interval; PA = Physical Activity.

For discriminant validity, the magnitude of correlation coefficients was similar between absolute PA and relative PA. For absolute PA, correlations with discriminant validation measures ranged from 0.04 (95% CI: 0.00 to 0.19) to 0.29 (95% CI: 0.15 to 0.50) and for relative PA, correlations ranged from 0.02 (95% CI: 0.00 to 0.12) to 0.24 (95% CI: 0.00 to 0.44). In general, comparisons of discriminant validity measures and PA questions led to correlations that indicated little to no association.

## Discussion

The absolute PA question had better test-retest reliability than the relative PA question. Paradoxically, evidence for convergent validity was stronger for relative PA compared to absolute PA. For both questions, results indicated evidence for discriminant validity. The relative PA question had the best combination of test-retest reliability, convergent validity and discriminant validity. Specifically, there was moderate agreement when this question was re-administered seven days later, fair to moderate or good associations when compared with indicators of physical function, and little to no associations when compared with measures hypothesized to be *theoretically not *related to PA. Although we were unable to evaluate the five-level form of the relative PA question, a previous study examining the validity of a similar question from the National Health Interview Survey (NHIS) found that very little was gained with the 5-level question compared to the 3-level question [12].

Indicators of physical function, often referred to as *indirect *measures of PA, have not been commonly used to evaluate the convergent validity of single-item PA questions in older adults, despite recommendations for their use [16,17]. One study, evaluating two different PA questions in older adults, examined convergent validity against indicators of health (i.e., health conditions such as heart attack, stroke, and diabetes). This study did not report any type of validity coefficients, making comparisons with our findings difficult [9].

Two other studies that evaluated an additional four PA questions in populations of older adults, examined validity by comparing questions with summary measures from PA recall questionnaires. In the first study, a PA question designed to be used as a screening question in primary care was evaluated in a population of older women [11]. This question, "As a rule, do you do at least half an hour of moderate or vigorous exercise (such as walking or sport) on five or more days of the week?", was compared to two summary scores from the New Zealand Physical Activity Questionnaire - Long Form. Results indicated moderate agreement (κ = 0.46 to 0.56). In the second study, three PA questions from the NHIS (job-related activity, main daily activity, and activity compared to peers) were compared with summary measures from a detailed PA question set [12]. The main daily activity question asked, "How much hard physical work is required in your main daily activity? Would you say a great deal, a moderate amount, a little, or none?" The activity compared to peers question, "Would you say that you are physically more active, less active, or about as active compared to other persons you age?", was also expanded to a 5-level question with the following response options: *a lot more, a little more, about the same, a little less, a lot less*. For participants 65 years of age or older, correlation coefficients ranged from 0.17 to 0.21 for the main daily activity question and from 0.24 to 0.28 for the activity compared to peers question. The validity results from the present study, in particular for the relative PA question, have been similar or better than previous studies of single-item PA questions in older adults.

At least two studies have evaluated test-retest reliability of single-item PA questions in older populations. In the first study, researchers found intraclass correlation coefficients (ICCs) ranging from 0.75 to 0.80 for two PA questions that asked regular exercisers about their frequency and intensity of activity [9]. Another study evaluated the test-retest reliability of three different PA questions (work PA, strenuous PA, and moderate PA) in a sample of participants from the Canadian Mulitcentre Osteoporosis Study [10]. The kappa statistic was 0.57 (0.47 to 0.68) for the strenuous PA question and 0.30 (0.23 to 0.37) for the moderate PA question.

Reliability results achieved in the present study for the relative PA question were similar or better than those reported by Nadalin et al. [10] but worse than those reported by Davis et al. [9]. Comparing the results in the present study to those reported by Davis et al. [9] is also problematic, since the PA questions evaluated in that study were only posed to participants who had already reported engaging in regular exercise.

Indicators of physical function have been used to evaluate the convergent validity of many PA recall questionnaires designed for older adults. For a number of the most well-known questionnaires, evidence for convergent validity is not substantially stronger than that obtained in this study; in fact, in some instances, the relative PA question evaluated in this study, performed better. For example, correlations between summary scores from the Community Healthy Activities Model Program for Seniors (CHAMPS) Physical Activity Questionnaire and various measures of physical functioning ranged between 0.10 and 0.54 [16,18-20]. For the CHAMPS Physical Activity Questionnaire and the Yale Physical Activity Survey, test-retest reliability was evaluated over a similar interval to this study (one to two weeks), and ICCs ranged from 0.55 to 0.79 [18,19,21].

The intent of both the absolute and the relative PA questions was to quickly and easily classify older adults by their activity level. Since specific details related to frequency, duration and intensity are not referenced within the relative PA question, this question will remain accurate for assessment even when PA recommendations for older adults are revised, such as was done in the United States in 2007 [22] and in Canada in 2011[23]. The relative PA question may also be less prone to recall errors, compared to the absolute PA question, since participants do not need to remember the duration or frequency of their typically performed activities.

It is known that in general, people tend to over-report PA levels [24]. In the self-reported health literature, it was noted that with increasing age, people tended to overestimate their health when comparing themselves to others or alternatively, they underestimated the health of others [14]. Thus, it is plausible that the participants in this study may have overestimated their PA, and perhaps to a greater extent when responding to the relative PA question. This should be kept in mind when interpreting the results of this study and when considering the merits of measuring PA using an absolute or a relative question.

Other limitations exist for the present study. Participants included in this study were Canadian veterans of World War II or the Korean War and their caregivers, a highly selected group of older adults. In addition, some of the validation measures were only available on participants in the study who had reported at least one modifiable fall risk factor. The Project to Prevent Falls in Veterans (PPFV) began as a randomly selected sample; however, only 13% of the original participants were included as part of the risk factor modification trial and a smaller percentage completed the second clinical assessment and the final telephone interview. As a result, it is likely that the participants included in this study are different than the general population of older Canadian veterans and their caregivers. Caution should be taken in generalizing results from our study to populations that may differ clinically and demographically.

The present analyses were done because we had data that allowed us to do these comparisons, but were not part of a validation study planned a priori. It is therefore possible that the modest validity correlations achieved may be partially due to the measures selected for validation. Since there is no widely accepted criterion of PA [24], we chose to evaluate the convergent validity of two single-item PA questions, by comparing them with indicators of physical functioning. We recognize, however, that capacity to perform PA does not equal actual performance. As a result, correlation coefficients indicating more than a moderate association may not be possible when using indicators of physical functioning as validation measures. A related limitation is that while some of the indicators of physical function were objectively measured performance-based outcomes, others were measures of self-reported functional ability. Self-reported measures can be affected by factors such as cognitive impairment and guessing among older populations [25]. Additionally, it would have been preferable if the indicators of physical functioning were measured at the same time as the PA questions. Even so, we hypothesize that any resulting bias is likely toward the null, indicating that correlations may have been stronger if these measures had been conducted closer in time.

## Conclusions

In large sample research, there is a trade-off between the intensity of measurement of a single variable and the comprehensiveness of all variables. In this study, a relative PA question had the best combination of test-retest reliability, convergent validity and discriminant validity. The magnitude of the reliability and validity coefficients achieved for this question are similar, and in some cases better, than those previously reported for other single-item PA questions evaluated in older adults. Reliability and validity results of many recall questionnaires for older adults have also not substantially exceeded the results obtained in this study. This simple PA question may be useful in studies of older adults where PA is not the primary focus, but a brief classification of activity levels is needed.

In this study, we have taken an initial step in evaluating convergent validity of a relative PA question using indicators of physical functioning as validation measures. Future research should evaluate convergent validity using other validation measures such as accelerometers and more detailed recall questionnaires. This question or other single-item questions cannot replace recall questionnaires or other direct measures of PA when resources are available or when study objectives require more comprehensive measures. In summary, this simple PA question may provide an alternative to researchers when lengthy PA measures, which increase both cost and participant burden, are not possible or necessary.

## Methods

### Participants

We used data from the PPFV, a fall risk-factor screening and modification trial. The PPFV was approved by the Research Ethics Board for Health Sciences Research Involving Human Subjects at the University of Western Ontario. Written informed consent was obtained from all participants.

In 2002, the PPFV began with screening questionnaires mailed to 3,000 addresses of older adults living in central or southwest Ontario, sampled randomly from the client list of Veterans Affairs Canada. To be eligible, persons had to be: i) a Canadian veteran of World War II or the Korean War *or *someone providing care for this individual; ii) living independently; and iii) able to understand and provide responses to a screening questionnaire. Caregivers were not proxy respondents for veterans but were recruited as full participants in the study. Questionnaires were received from 1,913 veterans and 1,398 caregivers, which corresponded to a 70% response rate for veterans. The response rate for caregivers could not be calculated since the number of veterans who had a caregiver was unknown.

Participants from the London and Windsor regions were eligible to enroll in a one-year risk factor modification effectiveness trial. In total, there were 348 participants who consented to be re-contacted and who had self-reported at least one modifiable risk factor for falling. These participants were randomized to either the *Specialized Geriatric Services *(SGS) group (n = 188) or the *Family Physician *group (n = 160). Participants in the SGS group made two in-person visits where they received comprehensive clinical assessments; the first clinical assessment was conducted at the start of the trial (CA1) and the second clinical assessment was conducted at the conclusion of the trial (CA2). The SGS group was evaluated with the interRAI [26] and an assessment tool developed specifically for the PPFV, the VCA (see Figure 1). Because participants in the *Family Physician *group did not receive geriatric assessments, they were not included in the present analyses.

The main analysis of the PPFV revealed no significant differences between randomized groups in regard to falls or injurious falls. Accordingly, data from the PPFV were analyzed as a prospective cohort study. Participants with no reported modifiable risk factors for falling (Zero-Mod group) formed an open study arm (n = 91). Most participants in this group also received CA1 and CA2; however, only the interRAI was administered to these participants. At the end of the trial, a telephone interview including two PA questions was administered to all study groups. This telephone interview was completed as soon as possible after CA2. Participants in the SGS and Zero-Mod groups, who completed both a CA2 and the telephone interview, made up the validity sample (n = 159). Additional details related to the formation of the validity sample are outlined in Figure 1.

A reliability sub-study of items in the telephone interview was conducted in a convenience sample from the PPFV. This sub-study evaluated test-retest reliability, which refers to agreement among measurements on the same participants at different time points [27]. Participants who completed the telephone interview were asked if they would be willing to be re-interviewed seven days later, by the same interviewer. This process continued until the target sample size for the reliability sub-study was achieved (n = 43). Additional details on the PFFV have been presented elsewhere [28,29].

### Self-report PA measures

The absolute PA question was developed for the PPFV. This question, "What best describes your activity level?" had three response options: *vigorously active for at least 30 min, 3 times per week; moderately active at least 3 times per week; or seldom active, preferring sedentary activities*. Participants were asked to select the response option that best described their typical activity level. The relative PA question is similar to two questions included in the 1985 NHIS in the United States [12,30]. This question, "Compared to other people your own age, do you think you are . . . " had five response options: *much more active, more active, about as active, less active, or much less active*. Due to small numbers in the two most extreme categories, this question was re-coded as follows: *much more active *and *more active *were collapsed to *more active, about as active *remained unchanged, and *less active *and *much less active *were collapsed into *less active*.

### Validation measures

Validation measures were taken from CA2 since these measurements were completed closest in time to the PA questions. The earlier version of the interRAI Community Health Assessment is a standardized assessment tool that is a subset of the Minimum Data Set for Home Care (MDS-HC) version 2.0 [26]. Reliability and validity of the MDS-HC has been previously reported in community settings [31,32]. The version of the interRAI used in the present study provided detailed assessment in the following domains: cognition, communication/hearing, mood/behaviour, social and physical functioning, continence, disease diagnoses, health conditions, preventive health measures, nutrition/hydration, skin condition, environmental/home safety, service utilization, and medications. An additional section provided assessment of risk factors for falling.

The VCA tool was developed under the guidance of a geriatrician and a physical therapist specializing in geriatric assessments. This instrument was designed to be administered by trained geriatric health care professionals to capture information related to fall risk, including: home environment risk, chronic disease, health status indicators, sensory function, mobility, continence, cognition, pain, footwear, blood pressure, balance, strength, range of motion, gait and medications.

Convergent validity is present if two measures believed to reflect the same underlying phenomenon correlate strongly [33]. Eleven indicators of physical function from the interRAI and VCA were selected as convergent validity measures. Seven of these indicators are objective performance-based measures of physical function and four are self-report measures of functional ability. PA questions and indicators of physical function were ordered such that higher scores indicated higher PA levels or better functioning. We hypothesized a priori that evidence for convergent validity would exist if PA questions positively correlated with indicators of physical function.

Discriminant validity indicates that two measures believed to assess different characteristics will have little or no relationship [33]. Measures hypothesized to be *theoretically not *related to PA were selected as discriminant validity measures, and we hypothesized correlations close to zero. Three objectively measured items and one self-report item from the geriatric assessments were selected for evaluation. See Table 3 for an overview of validation measures and corresponding response options.

**Table 3:** Convergent and discriminant validation measures

Validation Measure	Response Options	**Assessment Tool**^**a**^
*Objective Measures (Convergent Validity)*		

1. Total score on Berg Balance Scale	Score range from 0 to 56	interRAI

2. Unsteady gait	▪ No ▪ Yes	interRAI

3. Gait (path)	▪ Straight without walking aid	VCA

4. Gait (trunk)	▪ No sway, flexion, use of arms, or walking aid ▪ Sway/Uses aid/Flexion/Spreads	VCA

5. Gait (abnormality)	▪ No ▪ Yes	VCA

6. Evidence of lower extremity weakness	▪ No ▪ Yes	VCA

7. Postural stability test (nudged)^b^	▪ Steady ▪ Staggers, grabs, catches self/Beings to fall	VCA

*Self-Report Measures (Convergent Validity)*		

1. Ability to walk 3 city blocks in last 3 days	▪ No difficulty ▪ Difficulty/Unable	interRAI

2. Difficulty with ordinary housework	▪ No difficulty ▪ Some/Great difficulty	interRAI

3. Limits going outdoors (fear of falling)	▪ No ▪ Yes	interRAI

4. Pain affects mobility	▪ No ▪ Yes	VCA

*Objective Measures (Discriminant Validity)*		

1. Vision	▪ Adequate ▪ Impaired/Moderately, highly or severely impaired	interRAI

2. Skin problems	▪ No ▪ Yes	interRAI

3. Evidence of hearing deficit	▪ No ▪ Yes	VCA

*Self-Report Measures (Discriminant Validity)*		

1. Home environment hazardous	▪ No ▪ Yes	interRAI

^a^Refers to the measurement tool that contained the validation measure

^b^Item from the Tinetti Balance Assessment Tool

Abbreviations: interRAI = interRAI Community Health Assessment (earlier version); VCA = Veterans' Comprehensive Assessment

### Statistical analyses

Test-retest reliability was assessed using the weighted kappa statistic along with 95% CIs [34]. Guidelines adopted for interpreting the strength of agreement for kappa values were as follows: less than 0.41 represents poor to fair agreement, 0.41 to 0.6 represents moderate agreement, 0.61 to 0.8 represents substantial agreement, and 0.81 to 1 represents almost perfect agreement [35].

Convergent and discriminant validity was assessed by correlation coefficients. Spearman's rho was used when the PA questions were compared with continuous validation measures whereas Cramer's v was used for validation measures that were categorical. For the Spearman's rho correlations, the Fisher z transformation was used to obtain 95% CIs [36]. Qualitative descriptors adopted for interpreting correlation coefficients were as follows: 0 to 0.25 represents little or no association, 0.26 to 0.5 represents a fair association, 0.51 to 0.75 represents moderate to good association, and greater than 0.75 represents good to excellent association [33]. All statistical analyses were performed using SAS v. 9.1.3 (SAS Institute Inc., Cary, NC, 2003).

## Abbreviations

CA1: First clinical assessment; CA2: Second clinical assessment; CI: Confidence interval; ICC: Intraclass correlation coefficient; InterRAI: interRAI Community Health Assessment (earlier version); MDS-HC: Minimum Data Set for Home Care; NHIS: National Health Interview Survey; PA: Physical activity; PPFV: Project to Prevent Falls in Veterans; SD: Standard deviation; SGS: Specialized Geriatric Services; VCA: Veterans' Comprehensive Assessment; Zero-Mod: Zero modifiable risk factors for falling.

## Competing interests

The authors (DPG, GRJ, GZ, MS) declare that they have no competing interests.

## Authors' contributions

DPG conceived of the study, participated in the design of the study, performed the statistical analysis and drafted the manuscript. GRJ participated in the design of the study and helped to draft the manuscript. GZ helped with the statistical analysis and helped to draft the manuscript. MS conceived the study, participated in the design and coordination of the study and helped to draft the manuscript. All authors read and approved the final manuscript.

## Pre-publication history

The pre-publication history for this paper can be accessed here:

http://www.biomedcentral.com/1471-2288/12/20/prepub
